# Phylogenetic Diversity of Animal Oral and Gastrointestinal Viromes Useful in Surveillance of Zoonoses

**DOI:** 10.3390/microorganisms10091815

**Published:** 2022-09-10

**Authors:** Anthony Michael Esposito, Michelle Marie Esposito, Albert Ptashnik

**Affiliations:** 1 Department of Biology, New Jersey City University, Jersey City, NJ 07305, USA; 2 Department of Biology, College of Staten Island, City University of New York, Staten Island, NY 10314, USA; 3 PhD Program in Biology, The Graduate Center, City University of New York, New York, NY 10016, USA; 4 DDS Program, NYU College of Dentistry, New York, NY 10010, USA

**Keywords:** metagenomics, virome, microbiome, zoonoses, periodontitis, pandemic prevention, COVID-19, surveillance

## Abstract

Great emphasis has been placed on bacterial microbiomes in human and animal systems. In recent years, advances in metagenomics have allowed for the detection and characterization of more and more native viral particles also residing in these organisms. The digestive tracts of animals and humans—from the oral cavity, to the gut, to fecal excretions—have become one such area of interest. Next-generation sequencing and bioinformatic analyses have uncovered vast phylogenetic virome diversity in companion animals, such as dogs and cats, as well as farm animals and wildlife such as bats. Zoonotic and arthropod-borne illnesses remain major causes of worldwide outbreaks, as demonstrated by the devastating COVID-19 pandemic. This highlights the increasing need to identify and study animal viromes to prevent such disastrous cross-species transmission outbreaks in the coming years. Novel viruses have been uncovered in the viromes of multiple organisms, including birds, bats, cats, and dogs. Although the exact consequences for public health have not yet become clear, many analyses have revealed viromes dominated by RNA viruses, which can be the most problematic to human health, as these genomes are known for their high mutation rates and immune system evasion capabilities. Furthermore, in the wake of worldwide disruption from the COVID-19 pandemic, it is evident that proper surveillance of viral biodiversity is crucial. For instance, gut viral metagenomic analysis in dogs has shown close relationships between the highly abundant canine coronavirus and human coronavirus strains 229E and NL63. Future studies and vigilance could potentially save many lives.

## 1. Introduction

**Figure 1:**
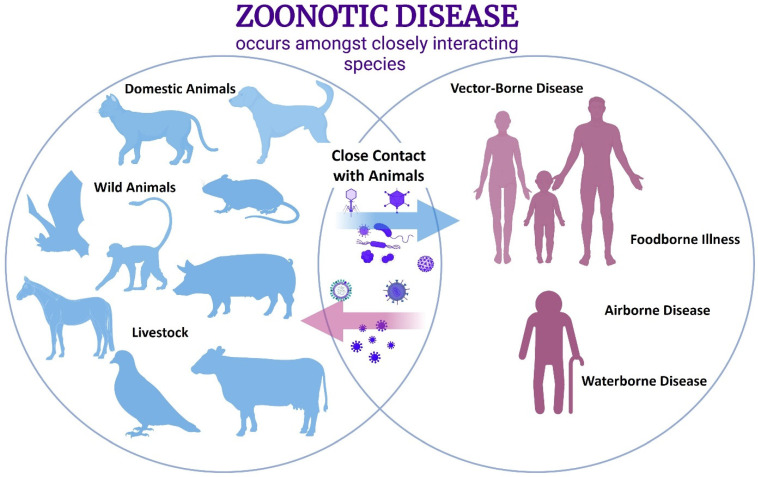
Zoonotic transfer of pathogens occurs when close contact encourages species-jumping transmission between animals—such as domestics, wildlife, or livestock—and humans. This transmission can be in the form of vector intermediates, ingestion of contaminated food or drinks, or the inhalation of droplets.

**Figure 2:**
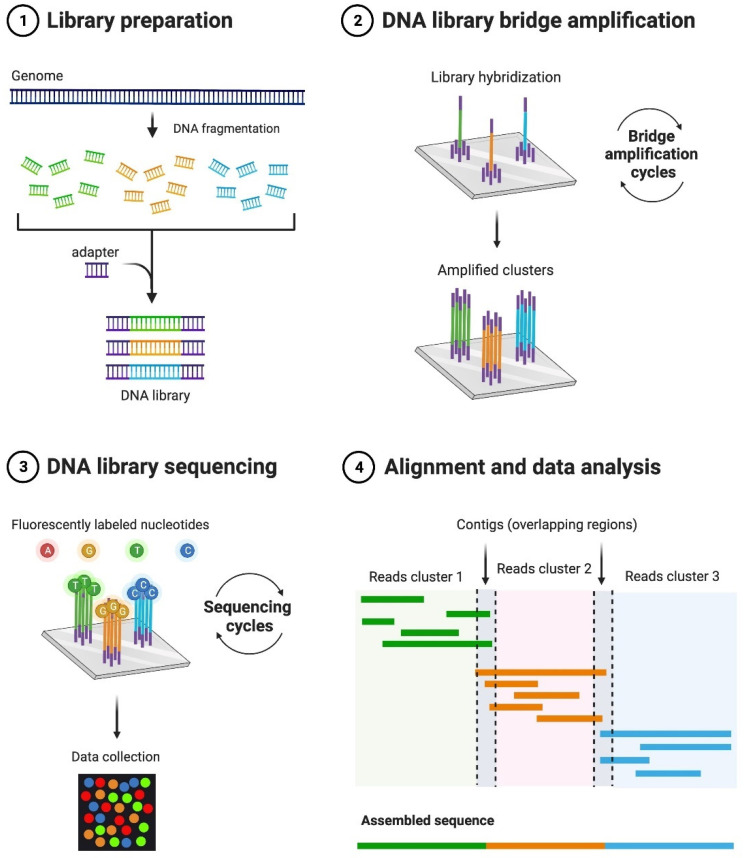
Schematic of the Illumina sequencing technology commonly used in metagenomic analyses of viromes.

Over the years, great emphasis has been placed on the bacterial microbiome [1]. In recent years, however, advances in metagenomics have allowed for the detection and characterization of more and more native viral particles also residing in the human body, as well as animal systems with the potential for zoonotic transfer [2]. Zoonotic simply means that a pathogen was residing in an animal reservoir but then jumped to a human host through close contact or interactions (Figure 1) [3]. The COVID-19 pandemic has demonstrated now more than ever the increasing need to identify and study animal viromes to prevent such disastrous cross-species transmission outbreaks in the coming years [4,5]. The majority of the global public does not recall ever experiencing a public health crisis to the level of the COVID-19 pandemic but, in fact, there have been other devastating pandemics during the previous century, including influenza outbreaks in 1918, 1957, and 1968 [6]. It is important to utilize the advances in technology since those eras to better prepare and prevent risks in the future. Previously, studies had to rely on highly time-consuming, labor-intensive, expensive techniques, such as reverse-transcription polymerase chain reaction (RT-PCR), which sometimes resulted in only low levels of data being acquired [7]. However, advances in metagenomic technology, such as Illumina sequencing (Figure 2), changed the game of virome analyses forever [7]. Metagenomics is both a set of research techniques and a growing field of research that can prove to be a powerful resource in biosurveillance, and can be combined with mathematical modeling to predict hotspots, high-risk locales or populations, and potential genetic variant emergence [6]. In this review, we focus on the significance of metagenomic analyses of animal and human digestive tracts—from the oral cavity, to the gut, through to fecal excretions—and how these studies can help us better understand zoonotic diseases.

## 2. Virome Analyses of Animals as Critical Tools in the Identification of Novel Viruses and the Surveillance of Known Viral Reservoirs

### 2.1. Wild Animals

One of the most unique animal reservoirs linked to several emerging or re-emerging outbreaks, including SARS (severe acute respiratory syndrome)-related coronaviruses, is the bat [8]. Metagenomic analysis of bats from China’s Yunnan Province has identified a novel bat-derived coronavirus, RmYN02, with 93.3% shared nucleotide identity at the complete viral genome level to the COVID-19 pandemic culprit—severe acute respiratory syndrome coronavirus 2 (SARS-CoV-2)—and 97.2% homology to the 1ab genome, making it the closest known relative of SARS-CoV-2 so far [9]. However, there is lower sequence similarity when viewing the receptor-binding domain (RBD), with only a 61.3% identity; thus, this virus may not be able to bind angiotensin-converting enzyme 2 (ACE2) receptors with the high affinity that was witnessed with SARS-CoV-2 [9]. The bat viromes of guano from five species of bats commonly found in Northern California have also been analyzed, uncovering several novel mammalian viruses, including novel coronavirus, bunyavirus, astrovirus, chaphamaparvovirus, nodavirus, densovirus, and circular Rep-encoding single-stranded (CRESS)-DNA virus sequences [10]. Although astroviruses and parvoviruses are significant as common enteric viruses associated with gastrointestinal disorders [11], the COVID-19 pandemic has shone a spotlight on the potential havoc that novel coronaviruses can also ignite. Screening sampled virome libraries with universal coronavirus PCR primers allowed for the identification of the novel alphacoronavirus in the Northern Californian bats, and although it shares high sequence homology with viruses found in bats in Florida and Brazil, it is not classified as being closely related to the problematic human SARS-CoV, SARS-CoV-2, or Middle East respiratory syndrome (MERS), all of which are defined as betacoronaviruses [10]. Interestingly, the bat virome also houses many insect viruses, such as a diversity of densoviruses, due to their robust insect diet [10]. Ultimately, bat guano has proven to be a robust source of information in potential pathogen surveillance through zoonotic virus monitoring, novel virus identification, and monitoring of prospective reverse-zoonotic spillover of SARS-CoV-2 into bat or other wildlife populations [10].

In addition to bats frequently being cited as a potential reservoir for historical pandemics and outbreaks, birds have also been a frequently targeted culprit, especially with regards to avian influenza (H5N1, “Bird Flu”) [12]. In fact, while the world was so focused on SARS-CoV-2, we mainly failed to notice that South Africa struggled with several outbreaks of H5N1 across their poultry farms during April and May 2021 [12]. To better understand the potential for novel zoonotic avian viruses, one study analyzed cloacal swabs from the digestive tracts of 3182 birds across China via Illumina MiSeq or HiSeq metagenomic sequencing and the Basic Local Alignment Search Tool (BLAST) to characterize the virome diversity [13]. Sampled birds included captive birds in zoos or on farms, as well as wild birds in natural reserves or parks [13]. The results demonstrated 707 viral genomes, with 469 of those being RNA viruses and 238 being DNA viruses, and with greater diversity observed in the wild samples versus the captive birds [13]. Amongst the observed viruses, similarities were observed to *Astroviridae*, *Picornaviridae*, *Caliciviridae*, *Hepeviridae*, *Coronaviridae*, and *Retroviridae*, indicating the potentials for cross-species transmission and genomic recombination [13]. In total, the virus samples comprised more than 87 different species, with many of them meeting the International Committee on Taxonomy of Viruses (ICTV) criteria to be classified as novel [13].

A 2019 study in Brazil also emphasized the significance of studying wildlife viromes, due to the strong wildlife–livestock–human relationship that exists in many places [14]. Furthermore, human activities, such as deforestation, have promoted a major shift in the distance of wildlife from humans, and have brought animals into greater contact with mankind [14]. Overall, it is believed that with these increasingly close contacts between humans and animals, epidemiological surveillance of animals’ susceptibility to viruses can be used as a means of early warning detection for preventing human health crises—especially since most emerging infections tend to be of zoonotic origins [14]. Between the span of January 2012 and September 2014, novel viruses from more than 17 virus families, totaling more than 73 new virus identities, were found in birds, and included viruses in the *Coronaviridae* family as the most numerous identified [15]. These findings, along with various other studies demonstrating the skyrocketing avian viral discoveries thanks to molecular advancements—especially Illumina sequencing Technologies—have led many people to realize the value of continuous global avian surveillance programs [15]. Brazil is known for its great diversity of fauna, so it serves as an excellent example of the robust animal virome data that can be obtained so quickly and easily with Illumina techniques [14]. A single study of Brazilian fauna found contigs from the *Adenoviridae*, *Anelloviridae*, *Caliciviridae*, *Circoviridae*, *Herpesviridae*, *Iridoviridae*, *Parvoviridae*, and *Poxviridae* virus families, totaling approximately 2000 viruses, which included novel species of adenovirus, anellovirus, parvovirus, and smacovirus [14]. Molecular and metagenomic analyses of such viromes can provide valuable information, as just recently it was found that smacoviruses—such as those found in the Brazil study—are not only residents of primate and human fecal viromes as suspects of gastrointestinal disease, but can also infect prokaryotes and, more interestingly, archaea [16]. The finding that these novel viruses can infect archaea suggests abilities to withstand extreme environments, which may then be of concern when looking to treat any infections eventually linked to them. This concerning observation further demonstrates the need to study viromes early rather than waiting until they become more problematic in humans.

### 2.2. Domestic Animals

High-throughput metagenomic sequencing using Illumina MiSeq and BLAST on healthy feline fecal samples from Portugal identified five eukaryotic enteric viruses [17]. The identified feline virome sequences included *Picornavirus*, *Astrovirus*, *Bocavirus*, *Rotavirus*, and *Picobirnavirus* [17]. It is of interest to note that these viruses were shed from healthy asymptomatic animals, and included a novel picornavirus proposed as the “*Sakobuvirus*” genus, which shares distant relatedness to the known human virus genera *Salivirus* and *Kobuvirus* [17]. Human viruses within both of these genera have been known to cause diarrhea and gastroenteritis, and have been observed in symptomatic as well as healthy individuals [17]. Rotaviruses are also commonly known to infect humans, and can be problematic in animals due to their potential for reassortment with human strains [18].

Illumina MiSeq was also utilized to study the feline enteric virome of cats from a single shelter in California [19]. The observed virome demonstrated some similarities to the Portugal study’s findings, including astroviruses, bocaviruses, and picobirnavirus, but the California study lacked picornavirus, and included additional viruses of the families *Coronaviridae*, *Circoviridae*, *Herpesviridae*, *Anelloviridae*, and *Caliciviridae* [19]. With zoonotic diseases on the rise worldwide, and with the potential for epidemic and pandemic levels of transmission, it is important to surveil any potential areas of human–animal interactions that are prime for cross-species transmissions. Although this California study took place prior to the COVID-19 pandemic, it is important to note the enteric virus families available for shedding in this study, as cats in shelters eventually end up with their forever homes after being exposed to high levels of circulating viruses in stressful shelter conditions, where their immune systems end up significantly weakened [19]. Those increased levels of stress have been shown to increase viral shedding, which can, in turn, increase the potential for unwanted transmission [19]. Mammalian viruses in the California shelter virome with high sequence similarity to known GenBank viruses included feline coronavirus, feline herpesvirus 1, feline calicivirus, feline norovirus, feline/human picobirnavirus, and feline panleukopenia virus [19]. The virome was also composed of approximately 1.2% viruses related to plant viruses, 0.2% viruses related to insect viruses, and 0.2% viruses related to fish viruses [19]. It should be noted that the Portugal and California samples both demonstrated a high percentage of astroviruses in the virome reads detected [17,19]. Astroviruses are known to cause diarrhea and GI (gastrointestinal) issues in a variety of species, with their main mode of transmission being fecal–oral [19].

In addition to native virome analyses in healthy cats, Illumina MiSeq genetic analyses were also of value in the analysis of an unexplained outbreak of vomiting and diarrhea across animal shelters in British Columbia, Canada [20]. Viral sequences identified in the fecal viromes included five main families of viruses—*Anelloviridae*, *Parvoviridae*, *Papillomaviridae*, *Polyomaviridae*, and *Caliciviridae*—demonstrating some overlap with the California and Portugal studies [20]. Three different bocaviruses were amongst the *Parvoviridae* family results, which is consistent with the heavy presence of these types of viruses in the Portugal and California studies as well [20]. However, most significant in this particular study is the fact that metagenomic analyses allowed for the discovery of a novel chaphamaparvovirus, which the authors named fechavirus [20]. This fechavirus was present in all vomit samples from the ill cats in the multiple shelters affected by the outbreak, suggesting a pathogenic role for the newly discovered virus and, thus, allowing for awareness of a future potential zoonotic pathogen, although this has not yet been found in humans [20].

In addition to cats, another domestic animal that is a prime candidate for cross-species transmission through close interactions with humans is the dog. Interestingly, a recent article published on the viral metagenome analysis of domestic dogs [21] shows many similar virome families that overlap with the studies identifying viruses in the cats of Portugal, America, and Canada [17,19,20]. Next-generation sequencing metagenomic gut analysis of 45 domestic healthy canines in China demonstrated the greatest abundance of the DNA virus families *Circoviridae*, *Parvoviridae*, and *Herpesviridae*, as well as the RNA virus families *Astroviridae*, *Coronaviridae*, and *Picornaviridae*, in the 63 virus families observed (31 DNA virus families and 32 RNA virus families) [21]. As with the California cat virome analyses [19], sequences homologous to known plant and insect viruses were also observed in the dog viromes [21]. Although it is interesting that the astroviruses and bocaviruses that were so abundant in the cat studies [17,19,20] also appeared to be heavily dominant in domestic dog samples, it is even more concerning that the canine virome revealed a coronavirus—closely related to the canine coronavirus circulating in China, USA, and Italy—in high abundance in all of the canine samples analyzed [21]. Even more worrisome with respect to the potential for cross-species transmission and potential future zoonotic pandemics is that the observed canine coronavirus B203 and B363 strains demonstrated close phylogenetic similarities to the human 229E and NL63 coronavirus strains [21]. As with the feline studies, metagenomic analysis of gut viromes was also used in canine studies to analyze dogs that were part of an infectious diarrhea outbreak that had occurred in Colorado [22]. As was observed with the feline multi-shelter outbreak of diarrhea [20], virome testing of dogs involved in a diarrhea outbreak also uncovered a previously unknown parvovirus that may have been to blame [22]. Although the parvovirus, tentatively named CachaV-1, was only present in two of the nine sampled animals, it was reasoned that many of the dogs sampled more than 10 days after the onset of symptoms were no longer in the viral shedding stage of transmission, which would have allowed for proper detection [22]. When statistics were then run on larger samples of healthy animals versus those with bloody diarrhea symptoms to detect the cachavirus DNA sequence, statistical significance was observed, and borderline association was concluded [22]. The virome of dogs with acute diarrhea versus healthy dogs was also analyzed in a study in Australia using shotgun metagenomics [23]. In these samples, bacteriophages were present in the highest percentage of the virome—especially the *Caudovirales* order and *Microviridae* family—but eukaryotic viruses of the *Coronaviridae*, *Parvoviridae*, *Reoviridae*, *Caliciviridae*, *Astroviridae*, and *Picornaviridae* families were also identified [16]. As in many of the studies that are discussed in this review, there was a strong prevalence of astroviruses in particular [23]. *Reoviridae* are most famous in humans for the genus of *Rotaviruses*, which are responsible for 30% of all diarrhea-related mortalities globally in children under the age of five years [24]. Although the presence of astroviruses and reoviruses was consistent with other studies of canine viromes, the most interesting suggestion from this study revolves around the high percentage of bacteriophages in the viromes of the diarrhea-plagued dogs. It was suggested that the large communities of bacteriophages may have disrupted the native bacterial microbiomes of the gut and, thus, lead to diarrhea; conversely, a change in bacterial composition leading to diarrhea may have resulted in the observed change in bacteriophage levels [23]. Either way, the findings of the Australian study demonstrate the potential value of conducting future studies to investigate viromes and bacteriomes at the same time [23]. It should also be noted that this study emphasized the role that age can play in viromes, as the highest affinity for some viruses—such as those of *Coronaviridae*, which can be fecally shed for up to 156 days—exists in younger individuals, such as puppies [23].

Although many people know the term “bird flu”, and associate bats with the theories behind the origins of the COVID-19 pandemic, one of the wild animals frequently neglected in everyday thought of animal reservoirs is the camel, seeing as they do not exactly roam within sight of the majority of global locations. It would be a great oversight to neglect to examine these animals in a discussion of zoonotic surveillance of interest in preventing outbreaks, considering that camels are known reservoirs of various coronaviruses, are factories of highly potent nanoantibodies with promising clinical applications, and were even credited as the culprits of the MERS-CoV (Middle East respiratory syndrome coronavirus) outbreak of 2012 [25]. Camels are not just wildlife roaming free in some regions, but also happen to be one of the major types of livestock in some locations, such as Kenya, putting tourists and camel handlers—including farmers, butchers, and traders—at risk of infections [26]. Despite being known for housing coronaviruses, fecal virome analyses have shown that camels also harbor robust reservoirs of highly diverse *Circoviridae* and *Picobirnaviridae* viruses [27]. As with many of the other animals described in this review, the study also demonstrated high abundance of *Picornaviridae*, *Parvoviridae*, *Astroviridae*, and *Hepeviridae* amongst the 7330 viral contigs assigned [27]. The clinical implications of these findings have not yet been well established, as even though some of the identified viruses—such as picobirnaviruses—have been found in ill children and immunocompromised patients, they have also been found in healthy humans, and it is important to note that these include viruses known to have the ability to infiltrate human cells [27]. Additionally problematic is the fact that even though people may not expect to be in close contact with camels, there are many ticks that can serve as an intermediary between their camel hosts and humans, with a wide array of tick-borne viruses [26]. Metagenomic analyses of the viromes of ticks infecting camels in Kenya revealed potential zoonotic pathogens known as Mbalambala tick virus, Bangali torovirus, Bole tick virus 4, and Liman tick virus [26]. The presence of zoonotic pathogens identified in these virome analyses is significant, as these tick-borne viruses have also been identified in virome analyses in other countries, such as Russia and China, so they are not only found in animals native to Africa or the Middle East [26].

### 2.3. Livestock Animals

Although most people think of dogs and cats when they envision daily human interactions with animals, farm animals also represent a large population of animals that pose a risk of potential zoonotic transfers. One of these animals that has been found to be a reservoir for various viruses is the pig [28]. Sequence-independent amplification, high-throughput sequencing, and metagenomic analyses of fecal viral contigs of asymptomatic East African farm pigs compared to GenBank database BLASTx sequences showed many contigs with no known similarities, but also many with high sequence similarity to known viruses [28]. The most robust similarities in the samples matched mammalian viral genera also described in this review in the wildlife and domestic animals sections, including *Astrovirus*, *Rotavirus*, *Bocavirus*, *Circovirus*, and *Kobuvirus* [28]. The less frequent contigs in the porcine fecal samples included one genus already listed in this review as being common in the viromes of domestic animals—*Picobirnavirus* [17,19]—while the others (e.g., *Sapelovirus*, *Pasivirus*, *Posavirus*, *Teschovirus* [28]) were not observed in the wildlife or domestic studies explored in this review. While some of these viruses are specific to pigs, such as *Teschovirus*, it is concerning that some are already known to be pathogenic to humans, such as *Astrovirus*, *Rotavirus*, *Bocavirus*, and *Kobuvirus* [29,30,31]. When metagenomics was also used to analyze the enteric viromes of pigs in the United States, similar main genera were observed in the highly diverse viral contigs, even though these pigs were not asymptomatic but, rather, were infected by porcine endemic diarrhea virus (PEDV) [32]. Kraken-algorithm-based genomic analyses demonstrated genera that included (in order of robustness) *Mamastrovirus*, *Enterovirus*, *Sapelovirus*, *Posavirus*, *Kobuvirus*, *Sapovirus*, *Teschovirus*, *Pasivirus*, and *Deltacoronavirus* [32]. It should be noted that all viruses found in the study of the diarrhea-afflicted farm pigs [32] belonged to four virus families found prominently throughout the wildlife and domestic studies as well: *Picornaviridae*, *Coronaviridae*, *Astroviridae*, and *Caliciviridae* [13,19,21]. Previous studies of healthy versus diarrhea-afflicted pigs in United States farms, such as in North Carolina, had the same results of an overwhelming presence of viral sequences (99% of the sequences in one study) belonging to the same four virus families of *Picornaviridae*, *Coronaviridae*, *Astroviridae*, and *Caliciviridae*, with only 1% of the sequences belonging to the DNA viruses *Circoviridae* and *Parvoviridae* [33]. These consistent findings, which range from over a decade ago to within recent years, are concerning, as each of these virus families has been shown to be prone to recombination, co-infection, and accelerated viral evolution, all of which are prime perfect storms for zoonotic spillover pandemic events [33]. Overall, analyses of diarrhea-afflicted and healthy pigs can be very valuable, as it has been demonstrated through multiple metagenomic surveillance projects that high standards in livestock industries practicing strict biosafety can help reduce pathogenic viral spread to not only the pig farms, but also to the humans whose lives have become so deeply interwoven with this invaluable livestock staple [34]. It would be especially valuable to emphasize surveillance in China, which represents more than 55% of the world total of annual pig slaughters as the largest global pig-farming industry, and has been the location of live markets suspected of cross-species transmission of outbreaks in the past [34]. It should be noted that pigs are already credited as host reservoirs known to spread problematic pathogens to humans, including hepatitis E, Nipah, influenza A (including the infamous H1N1 swine flu pandemic), and Japanese encephalitis viruses [34,35,36,37,38].

Interestingly, the pattern of dominant virome families in farm animals is not restricted to pigs, and was even demonstrated in a recent study using metagenomic analyses of healthy and diseased broiler flocks in the poultry industry [39]. In that study, *Parvoviridae*, *Astroviridae*, *Picornaviridae*, *Caliciviridae*, *Reoviridae*, *Adenoviridae*, *Coronaviridae*, and *Smacoviridae* were all observed in the broiler flocks [39], with *Adenoviridae* and *Smacoviridae* being the only two of these families of viruses that were not heavily observed in the other wildlife studies mentioned so far in this review. The lack of Smacoviridae in the other studies could, however, simply be due to the fact that it is a newly classified category thanks to metagenomic approaches, and was not added to the International Committee on Taxonomy of Viruses (ICTV) until 2018 [40]. This study also demonstrated *Chaphamaparvovirus* in all flocks [39], which are viruses that were also already mentioned in relation to bat viromes [10] and healthy cat viromes [20]. The study on broiler fowls corresponds with the findings of other studies in which diarrhea- or illness-stricken and healthy animals were both found to have abundant *Chaphamaparvovirus* in their viromes, including a recent study of ducks in Australia [41], as well as nephropathy-inflicted mice in Australia and America [42] and diseased dogs in America [22]. A novel *Chaphamaparvovirus* was also recently found to be the etiological agent responsible for several devastating high-mortality hepatitis outbreaks in French pheasants [43].

## 3. Human Virome Metagenomic Analyses Also Provide Valuable Information

### 3.1. Human Oral Virome Significance

**Figure 3:**
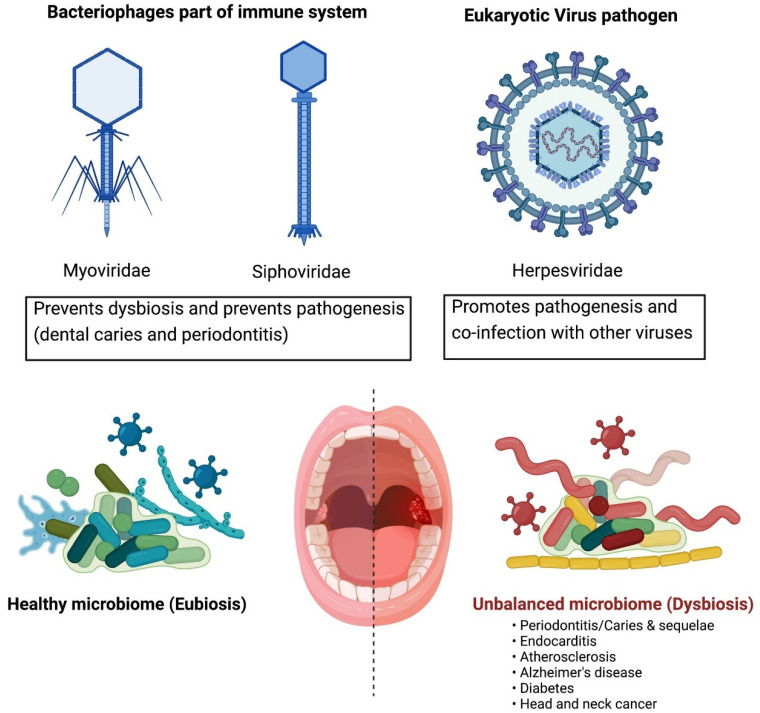
Dominant oral cavity viruses, including lytic bacteriophages (e.g., Myoviridae and Siphoviridae) that aid in immune protection, as well as pathogenic eukaryotic viruses (e.g., Herpesviridae) that threaten dysbiosis.

Although the COVID-19 pandemic made the world view viruses as the villain, metagenomic analysis of viruses native to the human body has provided a different perspective to these misunderstood microbes. Whereas understanding animal virome compositions may help in pandemic prevention surveillance, understanding the human virome may help us to maximize our immunity and to better understand how imbalances in normal microbial flora (dysbiosis) can lead to medical crises, including neurodegenerative diseases [44,45], heart diseases [46], diabetes [47], and various cancers, including head and neck cancers [48,49] (Figure 3).

Similar to the more heavily studied bacterial microbiota of humans, the viral microbiota appears to exhibit great diversity when different parts of the body are analyzed [50]. The human oral microbiome (HOM) is of great scientific significance as the second most robust microbial collection in the body other than the normal gut flora, and while bacteria have always been the main focus, more and more attention is now being devoted to the non-bacterial components of the HOM, such as viruses [51]. In the oral cavity, broad-range 16S rRNA PCR amplification and pyrosequencing has revealed an abundance of bacteriophages appearing to dominate the salivary viral microbiota [50]. BLASTn analyses allow for the determination of the conservation of homologs amongst subjects, and have demonstrated extensive homology in identified viral sequences [50]. Analyses of those viral communities have demonstrated that the metabolic profile of the oral viral ecosystem includes nucleic acid metabolism and virulence pathways, but most lacked known metabolic homologies [50]. The identification of prevalent virulence factors in viral contigs of the oral cavity has demonstrated that, as with the animal virome analyses, characterization of the human viral microbiota can also help us to understand their role in human health [50]. Interestingly, the viral contigs identified in the oral cavity differed from the viral analyses of stool and respiratory systems, and seemed to demonstrate a large proportion of lysogenic bacteriophages that may be involved in interactions with the bacterial microbiota—particularly *Veillonella*, *Streptococcus*, and *Megasphaera* [50]. Multiple studies have demonstrated similar composition of the oral virome, with *Actinomyces* viruses and *Streptococcus* phages being found at high frequencies [50,52]. It has been suggested that immunoglobulin A (IgA) may play a role in the diversity of the oral microbiota, as it is the dominant mucosal antibody, and lower amount of microbes are observed in its absence [52]. It is believed that this role may be due to IgA’s glycans serving as a carbon source for the microbiota, although deficiency studies show that commensal microbiota are resilient enough to overcome some IgA deficits [52]. The presence of oral bacteriophages as a mechanism of maintaining bacterial homeostasis and preventing dysbiosis is significant in the prevention of pathogenesis—particularly of those diseases seen in the dental field, such as caries and periodontitis [53]. With more light being shed on the overwhelming presence of bacteriophages in the oral cavity and their influences on the biofilms in those crevices, phage therapy is now considered a potential area of study for the management of dental plaque and periodontal complications [54]. It should be noted that periodontal interventions and care concern so much more than just the mouth. In fact, periodontal diseases in susceptible hosts have been linked to various other ailments of organs, including the heart, brain, kidneys, liver, and reproductive system, as well as systemic complications and cancers [55]. Diseases now linked to periodontal issues include Alzheimer’s disease, heart disease, obesity, pregnancy complications, and diabetes [55].

One of the most recent diseases in which metagenomic virome analyses, including comparisons to the prokaryotic microbiome, have helped advance clinical knowledge is hand, foot, and mouth disease (HFMD), which is known for its rampant spread through child populations [56]. HFMD presents with a variety of symptoms that range from mild rashes all the way to neurological complications and fatalities [56]. While it was already known that HFMD is frequently caused by enteroviruses and coxsackieviruses, metagenomic analyses of saliva in symptomatic, asymptomatic, and healthy individuals revealed significant alterations in oral virome composition in symptomatic samples [56]. The most significant differences observed in virome composition analyses of symptomatic HFMD samples were in streptococcal species correlated with enterovirus A levels, and in coxsackie A5 viruses in asymptomatic saliva versus coxsackie A6 viruses in symptomatic samples [56]. Although asymptomatic cases or samples are commonly neglected in favor of focusing on symptomatic conditions, it should be noted that the high frequency of recombination in viral particles makes dominant asymptomatic strains potential reservoirs of future pathogenesis and outbreaks [56].

Another disease of oral metagenomic interest is SARS-CoV-2 (the causative agent of COVID-19). Metagenomic whole-genome sequencing (WGS) to better categorize simultaneously oral bacteria, fungi, and viruses in COVID-19 patients has helped to demonstrate the role that oral viruses may play in either promoting or hindering infections [51]. It is suggested that COVID-19 patients tend to have oral microbiome dysbiosis, with a noticeable co-infection of other viruses present in their oral virome, such as an increased presence of the herpes simplex I (HSV-1) and Epstein–Barr virus (EBV) herpesviruses, which may contribute to pathogenesis and decreased efficiency of immune responses [51]. EBV, which was found to be part of the non-bacterial HOM of approximately 30% of COVID-19 patients versus only 5% of controls, as well as reactivation of alphaherpesviruses (e.g., HSV-1, varicella-zoster virus), was associated with more significant complications of COVID-19, including fatalities [51].

In exploring the prevention of pathogenesis, it is also of great importance to understand what shapes the diversity or composition of these oral virus communities, as this viral composition then has the power to shape the bacterial communities within the human host. Analysis of oral viruses has uncovered interesting patterns with regards to homeostasis and microbial community ecology, including the findings that the viromes of unrelated household contacts are significantly more similar than those of non-household contacts, and that oral virome compositions are associated with the sex of the hosts [57]. These analyses demonstrate that shared environmental reservoirs and hormones play a determining role in the composition of oral virome ecology [57]. In saliva samples, *Streptococcus*-infecting phages have been shown to be dominant [58]. Oral community ecology studies have also demonstrated that lytic viruses, such as *Myoviruses*, dominate the subgingival crevice in periodontal disease, whereas lysogenic phages, such as *Siphoviruses*, inhabit the supragingival biofilms and planktonic saliva, and are the standard in healthy subjects [57]. Lytic viruses are more potent driving forces of bacterial diversity, whereas lysogenic viruses integrate into the host and maintain a more stable host existence, thus not causing as much fluctuation in microbial ecology as much [59]. It will be of value if future studies of viromes bear this concept in mind and continue to categorize viral ecologies based on lytic versus lysogenic predominance to better determine the impact these viruses can have on host microbiomes and, thus, disease states. Overall, the heavy dominance of lytic and lysogenic bacteriophages—*Myoviridae*, *Siphoviridae*, and *Streptococcus* phages, as well as the eukaryotic viral *Herpesviridae* (Figure 3)—in oral cavities has been observed not just in one location of human samples, but rather in varied geographic locations [60]. This geographic uniformity is intriguing, as the oral cavity is known for being subjected to a vast variety of fluctuating factors that include diet (including probiotics and prebiotics), medications, smoking and alcohol use, and dental hygiene practices, as well as interpersonal activities, such as kissing and oral sex [60].

### 3.2. Human Gut Virome Significance

**Figure 4:**
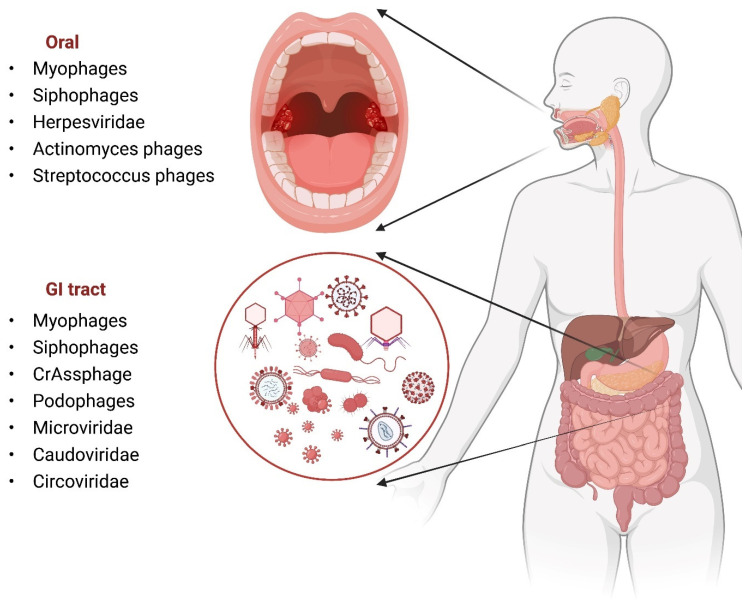
Oral and GI viromes share some common dominant members, but greater overall diversity is observed in the GI virome compared to the oral virome.

The gut microbiome has become increasingly well studied in the past few decades, correlating microbiome changes with a variety of diseases and overall human health. Less well-studied is the gut virome, which interacts with both the microbiome and host cells. Due to the abundance of bacteria in the gut, it is not surprising that bacteriophages make up the majority of the gut virome [61,62]. The first published metagenomic analysis of uncultured viruses focused on human feces from a single individual (a 33-year-old healthy male), and found that the majority of sequences identified were novel at the time [63]. The sequences that did match known viruses matched *Siphophages*, *Podophages*, and *Myophages*, which were also heavily dominant in oral viromes (Figure 4) [57]. More recent metagenomics analysis of fecal viruses in 10 healthy individuals found that crAss-like (cross-assembly phages), *Microviridae*, and *Siphoviridae* were the most prevalent [62]. A common theme in metagenomics analysis of gut viromes is the extremely high percentage of previously unclassified virus and virus-related sequences (frequently referred to as “viral dark matter”) that do not match other sequences currently in databases [64]. In addition, studies comparing the viromes of multiple individuals have found that the gut virome is individual-specific, varying greatly between individuals [62,65].

A recent study assembled and analyzed a database of 2697 human gut metagenomes (human Gut Virome Database) consisting of data from studies in five different continents in order to find any trends in individual gut viromes [66]. This database contains over 30,000 unique viral populations from individuals of a wide range of ages, including healthy individuals and individuals with a variety of health conditions, such as irritable bowel syndrome (IBS), inflammatory bowel disease (IBD), diabetes, and HIV infection. It was found that the gut virome varied greatly between individuals. There was no single viral population that could be found across all individuals, and the most ubiquitous viral population was only present in 39% of individuals [66]. The strongest trend that was observed was the age-dependent variation in viral populations in the gut viromes of healthy Western individuals. Infants and adults had the greatest richness of viral populations, while the crAssphage was not detectable in infants but was significantly more abundant in adults.

As is the case with the microbiome, changes in the human gut virome have been observed in a variety of diseases, such as inflammatory bowel disease (IBD) and irritable bowel syndrome (IBS). Individuals with Crohn’s disease (CD) and ulcerative colitis (UC) showed a significant expansion in caudovirus populations, with a concurrent decrease in bacterial diversity [67]. While this trend of caudovirus expansion was similar for CD and UC, the specific viruses responsible in each case were different, suggesting that the virome is specific for each of these diseases. These changes in phage populations may play a role in the dysbiosis that occurs in these individuals, but the exact relationship has yet to be fully determined and understood. Altered gut virome taxonomy has also been found in obese patients, with even greater alterations in those with type 2 diabetes [68]. Among the altered populations were the *Myoviridae* and *Siphoviridae*. *Streptococcus* phages, *Enterococcus* phages, and *Pseudomonas* phages were all found to have altered levels in obese individuals with type 2 diabetes when compared to healthy individuals, suggesting that the effects on the specific host bacteria of these phages may contribute to these diseases [68]. In addition, it was recently found that herpesviruses are also associated with obesity. In a serological profiling comparison of lean vs. obese Qatari adults and children, it was found that adults infected with HSV-1 were significantly more likely to be obese, although this relationship did not exist in the children in the study [69]. Celiac disease has also shown specific alterations in the gut virome, but these studies primarily show effects on the pediatric virome, which is discussed below in more detail [64,65,66].

As mentioned previously, the gut virome, along with the microbiome, can vary with age; therefore, analysis of viromes in specific age groups is warranted. Children with IBD (i.e., UC and CD) were found to have higher ratios of caudovirus populations compared to healthy controls [70]. This is consistent with what has been found in adult patients with IBD [67]. As is the case for adults, it is unknown whether these alterations to the virome play a direct role in IBD, or an indirect role due to their effects on the microbiome [71]. It has also been found that exposure to enterovirus resulting in detection of enterovirus in the stool of children aged 1–2 years is associated with a higher risk of developing celiac disease (CeD) later in life, while adenoviruses were inversely related, suggesting a possible protective role. Specifically, the risk was higher in individuals who had a higher gluten intake and had a genetic predisposition for CeD [72]. Another study focusing on children between the ages of 7 and 16 years found that individuals with CeD had significantly higher levels of human polyomavirus 2, enterobacteria phage mEpX1, and enterobacteria phage mEpX2 compared to healthy individuals. The aforementioned adenovirus effect was not observed, which may be due to the age, demographic, or methodological differences between the studies [73]. For children with diabetes, one study found that lower levels of Circoviridae-associated sequences and lower levels of virome diversity were correlated with an increased risk of type 1 diabetes [74]. Two other studies, however, did not find differences in the viromes of children with type 1 diabetes, but these discrepancies could again be due to differences in demographics and methodology [75,76].

A common trend among most virome studies is the high percentage of bacteriophages compared to eukaryotic viruses (Figure 4). For this reason, it is believed that the virome is tightly linked to the microbiome, and that alterations in one will correlate with alterations in the other. Infant monozygotic twins (less than 2 years old) have been shown to have strong gut virome and microbiome similarities, but at this stage both the virome and the microbiome are still dynamic [77]. The dynamic nature of the infant virome and microbiome means that dramatic changes in the composition of the virome and microbiome will occur as the individual ages, with less dramatic changes occurring in adulthood. It is believed that the phage–bacteria relationship, with its predator–prey dynamic, shapes the adult virome and microbiome as individuals mature and their diet and environment change between twins. A study of adult monozygotic twins found that the richness and diversity of the gut microbiome and virome of an individual were correlated with one another [78]. The study investigated 21 adult monozygotic twin pairs, finding a wide range of concordance amongst the pairs, with some having the same levels of concordance as dizygotic pairs. However, it was found that a high concordance of viromes was strongly associated with a high concordance of microbiomes, although it is difficult to determine the exact cause-and-effect relationship between the two.

Although many studies focus on bacteriophages in the gut virome, viruses that infect the eukaryotic host cells can also affect the microbiome and virome of individuals. Infection with HIV has been shown to be associated with adenovirus expansion in the gut [79]. This was also associated with alterations to the gut microbiome, possibly due to reduced CD4 T-cell levels. The more recent COVID-19 pandemic has also shown the effects of host infection on the gut virome and microbiome. While the alpha and beta diversity of gut viromes in COVID-19 patients did not differ from those of healthy individuals, some phages—such as Microviridae and Inviridae—showed greater abundance in COVID-19 patients [80]. This, as expected, was also associated with dysbiosis of the gut microbiome. Although the gut virome has only recently begun to be characterized and understood, it is already apparent that it plays a major role in affecting the gut microbiome and overall gut health. Hopefully, future studies will more specifically define the role of the gut virome in health and disease.

## 4. Conclusions

Even before the devastating COVID-19 pandemic, studies recognized that most pandemics start from cross-species transmission of viruses originating in animals [81]. Emergent and re-emergent viruses that have jumped to humans from animals with devastating consequences in recent times include SARS in 2003, MERS in 2012, H5N1 flu in 2003, H7N9 flu in 2013, H1N1 flu in 2009, Zika in 2016 [82] and, more distantly, the flu pandemics of the 20th century—Spanish flu in 1918, Asian flu in 1957, and Hong Kong flu in 1968 [83]. With zoonotic infections on the rise due to ecological, behavioral, and socioeconomic shifts in the world, it is important to develop new and effective methods for predicting and preventing such problematic transmissions [81]. Although it is difficult to predict which exact viral strain will be the cause of the next major worldwide outbreak, modern technologies can help identify and map the trends in the origins or spread of both novel and pre-existing strains [81].

Of particular value has been the emergence of advanced metagenomics technologies that allow for the full screening of various zoological viromes, spanning from the animals in our daily lives as domestic pets, to the animals encountered through farming, or even to the vast wildlife existing around us [84]. By metagenomically screening these viromes, humans can not only identify the predominant strains of various well-known viruses that exist in each geographic region, but can also identify and characterize novel viruses so that if one ever emerges as a cross-species threat, the development of diagnostics, treatments, or vaccines will be a much quicker process. Furthermore, pandemic prevention methods and screening techniques can even allow populations to distinguish more threatening pathogenic microbes from their more harmless counterparts through analyses of their molecular sequence potential, such as the key proteins that they are capable of producing [81]. If surveillance systems are enacted at the wildlife, livestock, and human population levels, with particular focus on geographic “hot spots” and strong intergovernmental collaborations, then pre-emptive actions would allow for containing potential threats at a local level prior to their rapid and more destructive spread beyond borders [82]. Even in our own review of this sampling of studies surrounding the metagenomics of viromes in wildlife, domestic animals, and farm animals, clear patterns emerge, with certain virus families demonstrating dominant presences in various species. Multiple novel viral sequences have also been identified through these techniques, including the novel bat-derived coronavirus RmYN02, which demonstrated such strong nucleotide homology to the culprit of the COVID-19 pandemic, SARS-CoV-2, that it has been labelled as the closest known relative of SARS-CoV-2 so far [9]. As coronaviruses (such as SARS-CoV-2) and influenza viruses are heavily prone to recombination events, it is important to pay close attention to such novel viruses identified in animal viromes, as recombination is a significant driver of viruses crossing species boundaries [85].

**Figure 5:**
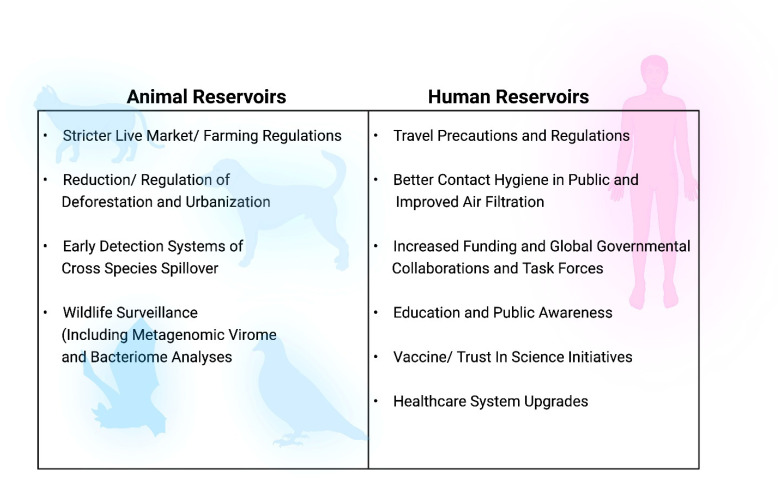
Suggested methods to reduce future pandemic risks from animal and human reservoirs.

Metagenomic surveillance of viromes is just one of many steps that can be combined to maximize the protection of the public and prevent future drastic public health situations, including pandemics as devastating as COVID-19. As zoonotic reservoirs pose one of the biggest threats to humans when analyzing the potential for pandemics, it is important to target animal protocols as well as human protocols when developing strategies to prevent public health crises (Figure 5). Some additional suggested means of protecting against animal reservoirs, beyond metagenomics, include stricter live market and farming regulations to reduce exposure to pathogens while those animals are still alive, as well as exposure of the meat after the animals have been butchered. Agricultural drivers have been found to be associated with more than 50% of zoonotic infections that have emerged in humans since the 1940s [86]. Live markets selling live animals and animal products are especially popular in low-income regions, and although they are a critical livelihood for these populations, they are frequently linked to historical outbreaks of coronaviruses and avian influenzas, with extremely close-contact exposure of wild and livestock animals to the retailers and customers in crowded conditions, with very poor sanitation levels [87,88]. These conditions are a perfect storm for not just zoonotic and food-borne infections, but also for the emergence of novel strains of viruses—particularly from cross-species recombination events [87,88]. Another major area beyond virome surveillance that can greatly improve the reduction in pandemic risks is unifying the efforts of governmental and international organizations to focus on public health initiatives and develop stricter regulations on deforestation and urbanization activities [89,90]. Deforestation and the destruction of habits has dramatically reduced biodiversity, created a shift promoting the expansion of species most likely to house dangerous pathogens that can cross to humans, and created increased contact between animals and humans that may not have encountered one another previously [89]. The impacts of deforestation can be seen in various populations, including rodents, bats, and primates, with zoonotic pathogen implications that include increased exposures to bacteria, viruses, fungal spores, and parasites, including helminths [91]. With deforestation, wild animals have ventured into domestic environments, and entered into increased contact not only with humans, but also with domestic animals, which then enter into close contact with humans [92]. In Sri Lanka, for instance, habitat destruction from deforestation has led to wild animals—including monkeys, boars, deer, pangolins, civets, porcupines, and elephants—suddenly roaming urban neighborhoods [92]. An example of the zoonotic repercussions of this population shift is that a substantial increase in wild boar numbers was then observed in those locations, as was an increase in numbers of *Dermacentor auratus* ticks previously considered to be very rare in urbanized environments [92]. The *D. auratus* ticks, eggs, or larvae that fell off the wild boars were then picked up by dogs, which served as an intermediate bridge to humans, who could then become infected with rickettsiae bacteria and Kyasanur forest disease (KFD), known to be transported by the *D. auratus* ticks [92]. Multiple other tick species capable of zoonotic spread of tick-borne infections and previously associated with forest-dwelling wildlife were also observed during this population shift [92]. Deforestation has also been implicated in increases in the incidence of malaria in nations across the globe [93], as well as the re-emergence of yellow fever virus in Brazil [94], to name but a few. Furthermore, the economic consequences of the COVID-19 pandemic, such as global poverty and increased food insecurity, have been suggested to compound the issue of deforestation by encouraging further illegal deforestation and bushmeat consumption—actions already heavily linked to the emergence of novel pathogens in humans [90].

While stronger surveillance and regulations of animal-related activities are critical to pandemic prevention, it is also important to focus on humans as well (Figure 5). Metagenomic analyses of our own human viromes, along with our microbiomes, can better develop our understanding of the interactions occurring under healthy versus ill conditions, which can then enable us to develop ways to maximize the good, healthy, normal flora microbes while minimizing the unhealthy, pathogenic ones. Of particular interest, as explored in this review, are the oral and GI viromes, but analyses should also be expanded to the other major tracts of transmission, including the respiratory tract and the genitourinary tract. Global virome projects could not only allow for advanced preparedness in terms of the early detection of potential threats, but could also improve diagnostic abilities and the development of therapeutics prior to waiting until it is too late and people are already overwhelmed by the pathogenic strains [95]. Proper pandemic and public health preparedness not only saves money, but can also save lives. It is important to consider all options early on, as it is not a question of “if” there will be another pandemic, but rather “when”, and our preparedness will determine how severe we allow these health crises to become.
